# The effects of enhanced external counter-pulsation on post-acute sequelae of COVID-19: A narrative review

**DOI:** 10.1515/med-2024-1067

**Published:** 2025-01-09

**Authors:** Jiecheng Huang, Yuxuan Fan, Yongshun Wang, Jingjin Liu

**Affiliations:** The Second Clinical Medical College, Jinan University, Shenzhen, Guangdong, China; Department of Rehabilitation, Tongji Hospital Affiliated to Tongji University, Tongji University School of Medicine, Shanghai, China; Department of Cardiology, Shenzhen People’s Hospital (The Second Clinical Medical College, Jinan University, The First Affiliated Hospital, Southern University of Science and Technology), Shenzhen, 518020, Guangdong, China; Department of Cardiology, Shenzhen Cardiovascular Minimally Invasive Medical Engineering Technology Research and Development Center, Shenzhen People’s Hospital (The Second Clinical Medical College, Jinan University, The First Affiliated Hospital, Southern University of Science and Technology), Shenzhen, Guangdong, China; Shenzhen Key Laboratory of Stem Cell Research and Clinical Transformation, Shenzhen People’s Hospital (The Second Clinical Medical College, Jinan University, The First Affiliated Hospital, Southern University of Science and Technology), Shenzhen, Guangdong, China; Department of Geriatrics, Shenzhen People’s Hospital (The Second Clinical Medical College, Jinan University, The First Affiliated Hospital, Southern University of Science and Technology), Shenzhen, Guangdong, China

**Keywords:** severe acute respiratory syndrome coronavirus 2, post-acute sequelae of coronavirus disease 2019, enhanced external counter-pulsation, anxiety, cardiovascular disease

## Abstract

Some of the millions of patients infected with severe acute respiratory syndrome coronavirus 2 (SARS-CoV-2) have developed new sequelae after recovering from the initial disease, termed post-acute sequelae of coronavirus disease 2019 (PASC). One symptom is anxiety, which is likely due to three etiologies: brain structural changes, neuroendocrine disruption, and neurotransmitter alterations. This review provides an overview of the current literature on the pathophysiological pathways linking coronavirus disease 2019 to anxiety, as well as the possible mechanisms of action in which an increasingly scrutinized treatment method, enhanced external counter-pulsation (EECP), is able to alleviate anxiety. SARS-CoV-2 triggers increased inflammatory cytokine production, as well as oxidative stress; these processes contribute to the aforementioned three etiologies. The potential treatment approach of EECP, involving sequenced inflation and deflation of specifically-placed airbags, has become of increasing interest, as it has been found to alleviate PASC-associated anxiety by improving patient cardiovascular function. These functional improvements were achieved by EECP stimulating anti-inflammatory and pro-angiogenic processes, as well as improving endothelial cell function and coronary blood flow, partially via counteracting against the negative effects of SARS-CoV-2 infection on the renin–angiotensin–aldosterone system. Therefore, EECP could promote both psychosomatic and cardiac rehabilitation. Further research, though, is still needed to fully determine its benefits and mechanism of action.

## 1 Introduction

Since December 2019, coronavirus disease 2019 (COVID-19), caused by severe acute respiratory syndrome coronavirus 2 (SARS-CoV-2), has had a huge impact globally. As of October 2023, >770 million infections and ∼7 million deaths have been reported worldwide (source: World Health Organization [WHO]). Some patients infected with SARS-CoV-2 develop new symptoms or sequelae after the disease, which can last for months or years; these manifestations are known as long covid, or post-acute sequelae of COVID-19 (PASC) [1], which has been defined by the WHO as a condition occurring in likely/confirmed SARS-CoV-2-infected individuals at least 3 months from COVID-19 onset, with symptoms lasting for ≥2 months, and unable to be explained with alternative diagnoses. The impact of PASC can be severe, affecting multiple organ systems, as demonstrated in a retrospective matched cohort study of 47,780 cases, which found that compared to the general population, those with acute COVID-19 had an increased risk for developing multiple organ dysfunction [2]. Furthermore, on top of damaging multiple organ systems, long COVID-19 could lead to negative alterations in psychological performance. Indeed, anxiety has been found to be one of the most common psychiatric symptoms among long COVID patients [3], as demonstrated in a meta-analysis of 132 studies, involving 9,320,687 patients, in which anxiety prevalence among PASC patients was 23% [4]. In line with this observation, a direct link between SARS-CoV-2 infection and anxiety has also been documented, further supporting the close relation between physical and psychological symptoms [5]. More specifically, stress, anxiety, and depression prevalence among the general population during the pandemic were, respectively, 29.6, 31.9, and 33.7% [6]; in particular, anxiety and depression prevalence in 2020 grew, correspondingly, by 27.6 and 25.6%, representing 76 and 53.2 million more cases [7]. PASC also has significant economic and social impacts, in that a prospective cohort study found that over a 3-month period, some patients had negative changes in their occupational status [8]. Considering that >65 million people suffer from PASC, the disease thus has a significant detrimental impact on employment and economic conditions.

In light of those findings, a number of potential strategies to alleviate the effects of PASC have been investigated, one of which is enhanced external counter-pulsation (EECP), a non-invasive assisted circulatory device developed in China. EECP was initially applied for treating various cardiovascular diseases (CVD), in which special airbags are used to arrange the calves, thighs, and buttocks of a patient in specified conformations. A computer then receives information regarding the diastolic and systolic phases of the heartbeat of the patient, based on their electrocardiogram R-wave, followed by inflation and deflation of the airbags in an orderly manner. More specifically, during diastole, the three groups of airbags are sequentially inflated, from the distal to the proximally located one, with a time difference of ∼50 ms between each inflation, leading to increases in diastolic pressure, as well as squeezing of the arterial and venous system of the lower half of the body. This results in more blood being supplied to the upper half of the body, in turn, improving perfusion of the heart, brain, and other important upper body organs. The sequential airbag inflation also simultaneously increases the venous return of the right heart, leading to improvements in heart volume per heartbeat and cardiac output. At the systolic phase, all airbags are synchronized to deflate, lowering the afterload and ejection resistance of the heart. This EECP procedure has also been found to have beneficial effects in treating COVID-19 sequelae in some studies [9,10,11], of which one of the most prominent sequelae is anxiety in PASC, which has increasingly become a topic of investigation. As anxiety has been demonstrated to be prevalent, and an important risk factor, among CVD patients [12], additional research regarding the underlying mechanisms behind anxiety and possible treatments to improve the quality of life among CVD patients would also be greatly beneficial for long COVID-19 patients. In fact, more and more research has been focused on how to decrease anxiety, rehabilitate sequelae, and help restore patient physical functions after PASC. In this review, we will describe the current literature on the pathophysiological pathways linking COVID-19 to anxiety, as well as the possible mechanisms of action in which EECP is able to alleviate anxiety and COVID-19 sequelae.

## 2 Mechanisms associated with PASC-associated anxiety

The main etiologies behind PASC-associated anxiety likely include brain structural changes, neuroendocrine disruption, and neurotransmitter alterations, all of which inflammation and oxidative stress (OS) play important roles. These three etiologies will be described below.

### 2.1 Brain structural changes

SARS-CoV-2 infection has been associated with neurologic manifestations, particularly in the form of brain structural changes; this has been documented in multiple case reports, but the underlying mechanisms have still not been fully elucidated. These mechanisms, though, may likely be related to SARS-CoV-2 neuro-invasiveness, -tropism, and -virulence [13,14], in which it has been found that compared to healthy controls, PASC patients with neuropsychiatric symptoms have significantly increased gray matter volume in various brain regions, including frontotemporal, cerebellum, hippocampus, amygdala, basal ganglia, and thalamus [15]. Additionally, a neuroimaging study has shown that patients with anxiety had structural and functional abnormalities in the prefrontal-limbic neural circuits, including prefrontal cortex, hippocampus, amygdala, insula, orbital frontal cortex, and cingulate gyrus, which may be closely related to anxiety onset [16]. In particular, the structures associated with the limbic system, comprising of different midbrain, diencephalon, and telencephalon components, are the most affected. This system is involved in numerous cognitive-associated processes, such as spatial memory, motivation, as well as emotional and social processing [17]. As a result, alterations in limbic system structure and function significantly correlate to psychiatric symptoms. This has been demonstrated in multiple studies, such as a meta-analysis of 320 studies, where patients with major depressive/anxiety disorders, and/or chronic pain had wide-ranging gray matter volume reductions in the insula and dorsomedial prefrontal/anterior cingulate cortices [18], as well as another meta-analysis of seven studies, where patients with anxiety disorders had significantly reduced spontaneous brain activity in regions such as the right putamen, right inferior orbitofrontal gyrus, and right temporal pole [19].

### 2.2 The contribution of inflammation and OS on neuroendocrine disruption

Inflammation and OS have been recognized as two major mechanisms contributing to COVID-19-caused anxiety (Table 1). In particular, an excessive inflammatory response has been observed to be triggered by the invasion of angiotensin-converting enzyme 2 (ACE2)-expressing immune cells with SARS-CoV-2. Indeed, ACE2 is widely expressed among human cells and has been identified as the host surface receptor for SARS-CoV-2 [20], whose spike (S) protein binds to it, facilitating viral entry. The excessive inflammatory response involves monocytes and macrophages producing copious amounts of inflammatory factors, such as interleukins (IL-1β, IL-6, IL-8, IL-18), tumor necrosis factor (TNF)-α, interferon (IFN)-γ, granulocyte colony-stimulating factor (G-CSF), monocyte chemoattractant protein-1 (MCP-1/CCL 2), macrophage inflammatory protein (MIP), and other chemokines. Elevated levels for those factors are characteristic of a “cytokine storm (CS)”, which has been documented to occur in some COVID-19 patients, such as in a meta-analysis of 28 studies involving 346 COVID-19 patients, in which 60% of COVID-19 patients had elevated IL-6 and 51% IL-8 in cerebrospinal fluid [21]. A 2024 meta-analysis, involving 103 studies and 5502 PASC patients, also found that those patients, versus control, had significantly higher levels of C-reactive protein (CRP) and 21 other cytokines; thus, PASC was associated with enhanced immune activation [22]. Additionally, PASC patients with neurological defects were found to have significantly higher IL-1β and IL-8 levels in a 2024 analysis of blood markers [23]. Furthermore, serum IL-6 and TNF-α have been particularly noted to be independent and significant predictors for disease severity and death [24], and PASC patients have been found to possess, mean IL-6 levels that were 29% higher in early recovery and 44% higher in late recovery stages, compared to controls [25]. This has been coupled with numerous other studies finding that COVID-19 patients had elevated IL-6 expression and decreased lymphocyte counts [26,27,28]. All of those observations were in line with Peluso et al. who found that persistent symptoms after COVID-19 may be associated with continued immune activation [25].

**Table 1:** The major mechanisms contributing to COVID-19-caused anxiety

Mechanism	Explanation
Inflammation [13]	Spike (S) protein of SARS-CoV-2 binds to ACE 2 expressed on the surface of host cells and mediates the entry of SARS-CoV-2 into the cells. As a result, SARS-CoV-2 could trigger CS
Oxidative stress [19,20,21]	High ROS and RNS levels stem from SARS-CoV-2 interactions with ACE2. After binding to ACE2, both ACE2 and SARS-CoV-2 are endocytosed by the host cell, leading to ACE2 downregulation, increases in Ang II and AT1R expression. AT1R promotes vasoconstriction, inflammation, and oxidative stress

SARS-CoV-2 = Severe acute respiratory syndrome coronavirus 2; ACE2 = angiotensin-converting enzyme 2; CS = cytokine storm; Ang II = angiotensin II; AT1R = angiotensin receptor type 1; ROS = reactive oxygen species; RNS = reactive nitrogen species.

Another process that has been linked with COVID-19-caused anxiety is OS, a state of altered redox homeostasis caused by psychological, physiological, or environmental stresses, which involves abnormally high reactive oxygen species (ROS) and reactive nitrogen species (RNS) levels, along with impaired antioxidant capacity and malfunctioning redox control *in vivo*. ROS and RNS are redox-active molecules with unstable oxygen and nitrogen molecules, possessing ≥1 unpaired electrons. They play important roles under physiological conditions, such as cell signaling and regulating redox homeostasis, but they have also been found to play important roles in SARS-CoV-2 pathogenesis and progression. More specifically, high ROS and RNS levels stem from SARS-CoV-2 interactions with ACE2, which have been found to play an important role in SARS-CoV-2-induced injury through multiple mechanisms: direct myocardial injury upon SARS-CoV-2 interaction with ACE2, as well as ACE2 downregulation and subsequent overstimulation of the renin–angiotensin–aldosterone system (RAAS) [29]. Notably, after binding to ACE2, both ACE2 and SARS-CoV-2 are endocytosed by the host cell, leading to ACE2 downregulation [30]. Lowered ACE2 expression, in turn, yields increase in angiotensin (Ang) II [31], which stimulates angiotensin receptor type 1 (AT1R) expression. AT1R promotes vasoconstriction, inflammation, and OS, particularly via Ang II binding to that receptor, leading to nicotinamide adenine dinucleotide phosphate (NADPH) oxidase activation and production of superoxide anions that result in mitochondrial damage and further ROS generation [32]. This ROS and RNS overproduction in SARS-CoV-2, owing to massive viral replication, leads to OS and damage to various host cell components, including proteins and mitochondria, yielding endothelial cell damage, disrupted lymphocyte and macrophage function, as well as exacerbated inflammation. Indeed, one study found that SARS-CoV-2-infected patients had higher OS biomarker levels compared to controls [33], demonstrating that OS is involved in tissue damage caused by SARS-CoV-2 infection. Moreover, another study conducted in 2023 found that compared to control, PASC patients were less able to resist oxidative damage, as indicated by lowered plasma total antioxidant capacity, glutathione peroxidase, and zinc levels [34]. Therefore, SARS-CoV-2 is associated not just with increased ROS and RNS production but also with lowered antioxidative defenses. As for PSAC-induced anxiety, it has been linked with elevated pro-inflammatory markers [35], such as in a cohort study showing that elevated CRP was observed in male patients with anxiety disorders [36]; another study found increased TNF-α in patients with anxiety disorders [37]. However, the specific mechanisms linking inflammatory factors to anxiety are still not fully elucidated and require further research.

**Figure 1:**
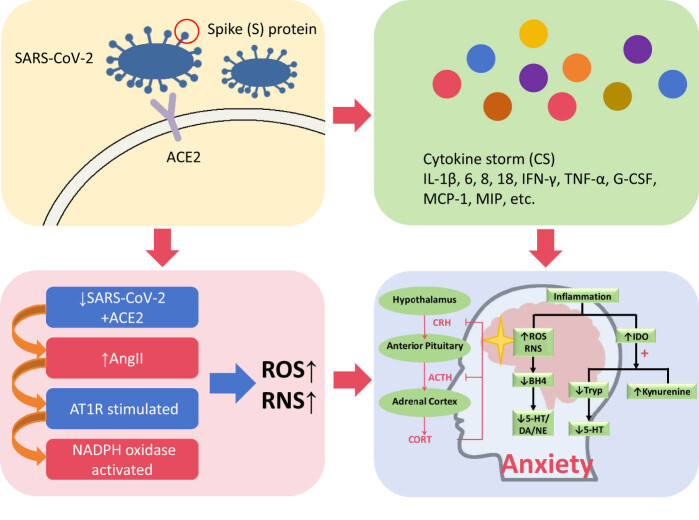
SARS-CoV-2 S protein binds to ACE2 on host cell surfaces, facilitating viral infection and triggering an excessive inflammatory response, entailing a CS comprising of IL-1β, IL-6, IL-8, IL-18, IFN-γ, TNF-α, G-CSF, MCP-1, and MIP. Additionally, SARS-CoV-2 binding to ACE2 lowers ACE2 expression levels, leading to increased Ang II levels, which subsequently increases AT1R and NADPH oxidase stimulation. The resulting increase in ROS and RNS, along with CS, yields alterations of hypothalamic–pituitary–adrenal (HPA) axis function and IDO activation, leading to increased anxiety, via cortisol dysregulation and inhibition of 5-HT production, owing to IDO conversion of tryptophan (Tryp) to kynurenine. Furthermore, ROS, RNS, and CS increases BH4 degradation, thereby negatively affecting 5-HT, DA, and NE synthesis. CRH = corticotropin-releasing hormone; CORT = cortisol.

The hypothalamic–pituitary–adrenal (HPA) axis, connecting the central nervous and endocrine systems, is an important structure in anxiety pathogenesis. It is involved in regulating hormones and the stress response. Upon stress and inflammation onset, the hypothalamus activates the HPA axis, via releasing corticotropin-releasing hormone, which subsequently stimulates adrenocorticotropic hormone (ACTH) release from the anterior pituitary gland; ACTH then acts on the adrenal cortex to induce cortisol synthesis and release. However, HPA regulatory mechanisms are altered under chronic stress, manifesting as sustained, elevated cortisol levels, and lowered sensitivity to cortisol feedback inhibition, resulting in decreased cortisol metabolism and higher plasma levels. Chronic cortisol dysregulation could lead to a variety of health problems, including anxiety [38]. Therefore, stress-induced neuroendocrine changes could serve as an important mechanism in the development of anxiety and other disorders [39]; the pathophysiological mechanisms are summarized in Figure 1.

### 2.3 Neurotransmitter disruption

Neurotransmitters have been documented to be important inflammatory mediators in the brain, in which peripheral inflammation could enter via three major pathways: humoral, neural, and cellular. The humoral pathway entails circulating cytokines entering the brain through “leaky” regions in the blood–brain barrier, followed by active transport into brain parenchyma via cytokine-specific saturable transport proteins, while the neural pathway involves cytokine signals being transmitted in the brain via afferent nerve fiber activation. The cellular pathway consists of activated microglia attracting peripheral inflammatory cells to the meninges and brain parenchyma. Therefore, cytokines and their associated signaling pathways play key roles in influencing anxiety-related neurotransmitter systems, as well as being involved in neurotransmitter synthesis, reuptake, and release; one prominent example is their activation of indoleamine 2,3 dioxygenase (IDO), which converts tryptophan, the primary amino acid for the neurotransmitter 5-hydroxytryptamine (5-HT), to kynurenine, thereby inhibiting 5-HT synthesis in the brain. Other mechanisms in which cytokines influence neurotransmitter activity are by facilitating the destruction of tetrahydrobiopterin (BH4) via increased ROS and RNS production, as it is extremely OS sensitive, resulting in its irreversible degradation to dihydroxyanthopterin. BH4 is an essential cofactor for tryptophan hydroxylase and tyrosine hydroxylase, both of which are rate-limiting enzymes for 5-HT, dopamine (DA), and norepinephrine (NE) synthesis; as a result, BH4 availability significantly impacts 5-HT, DA, and NE availability [40]. Furthermore, inflammatory cytokines increase the transporter protein expression and function for serotonin, DA, and NE, as well as upregulating neurotransmitter/precursor re-uptake and lowering their release. It should be noted that other neurotransmitter systems, such as gamma-aminobutyric acid and acetylcholine, may also play a role in anxiety development, though further investigation is needed to fully clarify the underlying mechanisms [41].

## 3 Mechanisms through which EECP treats COVID-19 sequelae

With respect to EECP, a number of recent studies, involving different patient populations, reported that it could aid in treating COVID-19 sequelae. One such study examined 51 patients with long COVID-19 and found that EECP treatment improved patient cognitive performances [9]. This was further reinforced by a retrospective analysis of 80 long-COVID-19 patients, of whom 38 had cognitive impairments and 42 did not; there, EECP substantially improved cognitive functioning in the impaired group [42]. Another COVID-19 sequela, which favorably responded to EECP, is fatigue, as observed in a systematic review of 20 studies, involving 5,629 PASC patients, where fatigue symptoms improved after receiving EECP [43]. This result was replicated for a single 38-year-old female with PASC symptoms after acute COVID-19, who had fatigue, headache, body aches, and shortness of breath during the acute infection phase. Even after most symptoms had been resolved, she still had periodic fatigue, headache, and “brain fog” for several months, which, however, improved with EECP treatment [10], thereby demonstrating the effectiveness of EECP in treating fatigue symptoms. Aside from psychological and mental improvements, EECP was also found to significantly improve cardiac function and exercise capacities, such as in one investigation where EECP, compared to baseline, yielded improvements in New York Heart Association functional classifications and left ventricular ejection fraction [11]. This finding was further supported by a study of 50 patients with refractory angina pectoris, where during the EECP treatment period, the average number of daily anginal episodes fell from 2.7 to 0.9, along with >70% of the patients having ≥1 grade reduction in their Canadian Cardiovascular Society classification, plus significant improvements in quality of life and exercise capacity, at 12 months post-EECP [44]. Cardiac functional and exercise capability improvements post-EECP administration were also found in a retrospective analysis of long-COVID-19 patients from seven different outpatient centers, where compared to baseline, they had improved scores on several functional tests after EECP, including the Seattle Angina Questionnaire and 6-min walk test [45]. Therefore, EECP has been noted to alleviate long covid symptoms and improve cardiac function, though it is still not fully clarified as to its mechanism of action in doing so. Nevertheless, some investigations have already been conducted regarding the mechanisms in which EECP exerts its beneficial effects, particularly in regard to hemodynamics and endothelial shear stresses [46]. These beneficial impacts, in turn, may serve as the basis behind EECP being able to alleviate PASC-associated anxiety (Table 2).

**Table 2:** Mechanisms of EECP on PASC-associated anxiety

Mechanism	Explanation
Hemodynamics [33,34,35]	EECP improves left ventricular hemodynamics by modulating aortic pressure, thereby increasing diastolic and decreasing systolic pressures, and subsequently increasing coronary blood flow velocity and pressure
	EECP reduces myocardial oxygen demand, in turn improving ventricular diastolic and systolic function, as well as increasing myocardial oxygen supply by promoting coronary artery collateral growth
ESS [38,39]	EECP induces increases in systemic ESS, which contributes to endothelial functional improvement. Additionally, ESS, through triggering multiple endothelial gene expression and signaling pathways, is able to inhibit abnormal cell proliferation, inflammation, and atherosclerosis; all of these processes aid in alleviating anxiety

EECP = enhanced external counter-pulsation; ESS = Endothelial shear stress.

### 3.1 EECP positively impacts hemodynamics and endothelial shear stress

EECP has been found to improve left ventricular hemodynamics by modulating aortic pressure, thereby increasing diastolic and decreasing systolic pressures, and subsequently increasing coronary blood flow velocity and pressure [47]. Additionally, EECP reduces myocardial oxygen demand, in turn, improving ventricular diastolic and systolic function [48], as well as increasing myocardial oxygen supply by promoting coronary artery collateral growth [49]. Indeed, a study of 50 patients found significant improvement in cardiac function classification and anxiety scale scores after just 1 course of EECP [50], while another study highlighted significant psychological improvements post-EECP among refractory angina patients [51].

EECP also induces increases in systemic endothelial shear stress (ESS), an important physiological stimulus for maintaining proper vascular endothelial function [52]. ESS has been noted to be a phenomenon associated with regulating the release of vascular endothelial factors, which, along with inhibiting inflammation, contributes to endothelial functional improvements. Therefore, EECP can affect OS and inflammation levels by increasing ESS, as previously documented in the literature [53,54]. Additionally, ESS, through triggering multiple endothelial gene expression and signaling pathways, is able to inhibit abnormal cell proliferation, inflammation, and atherosclerosis [55]. This was observed by Casey et al., where 35 h of EECP treatment increased both overall and local ESS, which subsequently lowered the expression levels of pro-inflammatory TNF-α and MCP-1 by, respectively, 29 and 20% [56]. As a result, EECP promoted anti-inflammatory and anti-atherosclerotic effects by increasing ESS and promoting NO release from endothelial cells; ultimately, all of these processes aid in alleviating anxiety.

### 3.2 EECP promotes cardiac and psychosomatic rehabilitation processes

EECP could also aid in cardiac rehabilitation, particularly in terms of recovery from SARS-CoV-2-linked myocardial injury and improving patient quality of life. This recovery is likely facilitated by EECP regulation of RAAS, along with stimulating increases in coronary perfusion and lowering endothelial injury. With respect to RAAS, ACE catalyzes Ang I conversion to Ang II, while ACE2 degrades both Ang I and II to, respectively, Ang 1–9 and Ang 1–7 [57]. SARS-CoV-2, though, lowers ACE2 expression in RAAS, resulting in increased ACE-catalyzed conversion of Ang I to Ang II. As Ang II binds to AT1R, this ultimately yields vasoconstriction and increased expression of cellular injury pathways. By contrast, EECP has been found in animal experiments to inhibit ACE expression, thereby counteracting against cardiomyocyte damage associated with the negative impact of SARS-CoV-2 on RAAS. This postulation is further supported by findings confirming that EECP increases diastolic coronary perfusion pressure and blood supply, with a meta-analysis showing a 150% increase in coronary flow velocity and a 28% increase in coronary flow after EECP [58]. Therefore, EECP augments myocardial perfusion via increasing coronary vasodilation, as well as promoting the angiogenesis of neoplastic collateral vessels in the myocardium. In particular, EECP stimulates the release of vasoactive factors, such as α-actinin, von Willebrand, and vascular endothelial growth factors (VEGF); in a randomized controlled study of 240 coronary artery disease (CAD) patients, VEGF1 and VEGFR2 expression levels were significantly higher in EECP versus control group after the 1-year follow-up period, and EECP-treated patients also had significantly improved endothelial function [59]. Additionally, EECP was associated with significant improvements in flow-mediated dilatation (FMD) among patients with left ventricular dysfunction, demonstrating that the treatment could significantly improve endothelial function [60]. Indeed, a controlled clinical study of coronary slow flow patients found significantly increased FMD and lowered CRP in EECP-treated, compared to control, suggesting that EECP is able to improve vascular inflammation and endothelial function [61].

Psychosomatic factors have been closely linked to heart disease occurrence and development, such as mental stress serving as an important trigger for myocardial ischemia in coronary heart disease. In fact, multiple studies have identified higher stress levels being associated with increased CVD risk [62], and strong correlations between abnormal psychosomatic states and adverse cardiac events [63,64,65]. Based on those findings, EECP may be able to provide beneficial effects, to a greater extent than conventional approaches, on psychological health factors.

### 3.3 Comparison with other treatments

EECP also ranks favorably with other non-invasive treatments for post-COVID anxiety, yielding comparable improvements to them. One of those other treatments is physical exercise, as found in a systematic meta-analysis of 8 randomized clinical trials, where compared to control patients who did not exercise, the exercise group had greater improvements in anxiety levels [66]. Another approach is prescribing anti-depressants, such as oxytocin and lithium salts [67], or herbal remedies, including valerian root [68] and passionflower extract [69], as well as kava-kava rhizome [70]. In particular, Silexan, a proprietary essential oil from Lavandula angustifolia, has been found in a meta-analysis of 13 studies by Kasper et al. to be effective in treating post-COVID-19 patients, particularly with respect to anxiety, owing to the therapeutic profile of Silexan overlapping with the range of psychiatric symptoms in those patients [71]. Additionally, an alternative electrical stimulation approach, transcranial direct current stimulation, has been found to be effective in alleviating fatigue [72] and anxiety [67]. However, it is still unclear precisely how effective these treatments are; future studies will thus be required to determine whether they are more or less effective than EECP and/or whether any complementary effects between these therapies are present.

## 4 Conclusion

This review summarizes the possible mechanisms underlying PASC-associated anxiety and explores the possible role and underlying mechanisms of EECP in serving as a treatment approach. SARS-CoV-2 results in anxiety via multiple pathways, including brain structural changes, neuroendocrine disruption, and neurotransmitter alterations, all of which inflammation and OS play important roles. EECP is able to counteract against the negative effects of SARS-CoV-2, by lowering inflammation, in turn promoting cardiac functional recovery, and ultimately decreasing patient anxiety. Therefore, EECP could serve as a potential treatment approach, in which it plays a dual role in psychosomatic and cardiac rehabilitation. Further research, though, is still needed to fully determine the benefits and mechanism of action for EECP, as few clinical studies have been conducted on EECP in treating long COVID sequelae. Therefore, future studies, involving multiple centers, double-blinding, and larger patient cohorts, should be carried out to further confirm the effectiveness of EECP for treating PASC-associated anxiety. These studies should also further identify the precise mechanisms/mediators underlying the anti-inflammatory and anti-oxidative effects of EECP, which ultimately counteract against the adverse brain structural changes, neuroendocrine disruption, and neurotransmitter alterations responsible for PASC-associated anxiety. Furthermore, these studies should compare the effectiveness of other treatment strategies, such as Silexan, to that of EECP for anxiety treatment, and formulate the optimal protocol for treating this disease, possibly involving additive or synergistic effects from a combination of those different strategies.
